# Medication Overuse Headache and Health-Related Quality of Life for Adults with Migraine in Saudi Arabia

**DOI:** 10.3390/jcm15082907

**Published:** 2026-04-11

**Authors:** Monira Alwhaibi, Ahad Almutairi, Salha Jokhab, Abdulrazaq Albilali

**Affiliations:** 1 Department of Clinical Pharmacy, College of Pharmacy, King Saud University, Riyadh 11149, Saudi Arabia; 2 Neurology Unit, Department of Medicine, College of Medicine, King Saud University, Riyadh 11149, Saudi Arabia

**Keywords:** migraine, medication-overuse headache (MOH), quality of life, migraine-specific quality of life (MSQ), headache disorders, patient-reported outcomes

## Abstract

**Background**: Migraine is a chronic illness that may impact the daily living and quality of life of affected individuals and might lead to excessive use of antimigraine medications. Quality of life in migraine patients is crucial, as it highlights the significant impact of migraines on daily activities, emotional well-being, and overall health. This study aims to assess the association between medication overuse headache and migraine-specific quality of life in migraine patients. **Methods**: A cross-sectional study was conducted at a neurology clinic in Riyadh, Saudi Arabia, from April 2025 to October 2025. Data about the quality of life were collected using the Migraine-Specific Quality of Life Questionnaire (MSQ). Medication overuse was identified using the International Classification of Headache Disorders, third edition (ICHD-3) criteria, and migraine severity was classified using the Migraine Symptom Severity Scale. Descriptive statistics were used to describe the study sample. Bivariate tests and multivariable linear regression were used to assess factors associated with MSQ. All statistical analyses were performed using SAS (ver. 9.4). **Results**: A total of 152 migraine patients were included, of whom 17.1% met the criteria for medication overuse headache (MOH). In bivariate analyses, MOH was significantly associated with lower Migraine-Specific Quality of Life (MSQ) scores across all domains (*p* < 0.001). Multiple adjusted linear regression confirmed MOH and migraine severity as the factors independently associated with reduced MSQ, with MOH associated with lower RR (β = –11.65), RP (β = –12.84), and EF (β = –16.23) scores (all *p* < 0.05). **Conclusions**: Medication overuse headache is common among migraine patients, affecting nearly one in six individuals in this study. It is strongly associated with increased migraine severity and a substantial reduction in quality of life across all domains. These findings highlight the critical need for early identification and appropriate management of medication overuse in clinical practice.

## 1. Introduction

Migraine is a chronic, episodic neurobiological disorder that significantly affects daily functioning and quality of life. It is characterized by recurrent attacks of moderate-to-severe headache, often accompanied by sensory hypersensitivity (photophobia and phonophobia), nausea and/or vomiting, and, in some cases, transient focal neurological symptoms known as aura [1]. Globally, migraine affects approximately 15% of adults and is more prevalent in women than in men [2,3,4]. In Saudi Arabia, its estimated prevalence reaches approximately 23% [5]. Migraine is recognized as the second leading cause of disability worldwide [6], and ranks among the leading causes of years lived with disability (YLDs) [4], profoundly affecting multiple aspects of life, including occupational, academic, social, and health-related quality of life [7,8]. Studies in Saudi Arabia have similarly shown that migraine significantly reduces quality of life [9,10]. Beyond its clinical and humanistic burden, migraine also imposes a substantial economic impact, increasing healthcare utilization and associated costs [11]. A systematic review of 13 studies further highlighted that chronic migraine places a significant financial burden on individuals, healthcare systems, and society, with direct costs, particularly hospitalization and medication expenses, representing the largest contributors [11].

Among the challenges faced by migraine patients is medication overuse, which can lead to a secondary condition known as medication-overuse headache (MOH) [12]. According to the International Classification of Headache Disorders 3rd edition (ICHD-3), MOH is defined as the occurrence of headache on 15 or more days/month in a patient with a known history of primary headache disorder, resulting from the regular overuse of symptomatic or acute headache medication (on 10–15 or more days/month, depending on the class of medication) for more than 3 months [1]. Globally, the prevalence of MOH in the general population ranges from 1.0% to 7.2% [13,14]. It is linked to significantly lower quality-of-life scores [15,16,17]. In Saudi Arabia, MOH prevalence was estimated to be around 4% from two studies conducted in Makkah and Qassim regions [18,19]. In Makkah, the prevalence of MOH among individuals who reported having experienced headaches was 4.5%, while only 18.7% of participants were aware of MOH as a potential consequence of frequent analgesic use [18]. Similarly, in Qassim, the prevalence of MOH was at 4%, with an almost identical awareness level of 18%, and paracetamol was the most commonly used analgesic in both regions [19].

Few studies in Saudi Arabia have investigated medication-overuse headache [18,19]. These studies have used online questionnaires disseminated via social media, increasing the risk of selection bias and limiting generalizability to the broader migraine population. Moreover, reliance on self-reported data without direct clinical evaluation may compromise diagnostic accuracy. In addition, none of these studies have examined migraine-specific quality of life (MSQ). To address this gap, the present study provides clinic-based evidence by evaluating the association between MOH and MSQ in patients with migraine, taking into consideration other independent variables that affect the MSQ in this population.

## 2. Methods

### 2.1. Study Design and Setting

This study was designed as an analytical cross-sectional study to assess associations between medication-overuse headache and quality of life outcomes among migraine patients. It was conducted in a neurological clinic in King Saud University Medical City and Dallah Hospital, Riyadh, Saudi Arabia, between April 2025 to October 2025.

### 2.2. Study Population

The study population included adult patients aged 18 years and older with a confirmed clinical diagnosis of migraine by a headache specialist and receiving abortive treatment. We excluded patients with headaches other than migraine, such as tension-type headaches, cluster headaches, cervicogenic headaches, or other secondary headaches due to underlying conditions (e.g., trauma, infection, or vascular disorders).

### 2.3. Data Collection

Data were collected using two methods to maximize participation and ensure comprehensive coverage of the target population. First, patients who attended the neurology outpatient clinic were invited to participate voluntarily during their clinical visits. The investigator approached eligible participants, and interviews were conducted in a private setting to ensure confidentiality and accuracy of responses. In addition, participants who could not be reached during clinic visits were contacted by telephone. During the call, the purpose of the study was explained, and verbal consent was obtained. Following consent, participants received a link to the study questionnaire via WhatsApp (Ver. 2.26.10.74), along with instructions on how to complete it. This mixed approach allowed for flexible data collection while maintaining participant privacy and data quality. It has to be noted that, all participants were identified and recruited during routine visits to the same neurology clinic. Due to variability in waiting times, some patients completed the study procedures on-site, while others were contacted later and completed them remotely (via WhatsApp) to avoid prolonging their time in the clinic.

### 2.4. Study Procedures

The survey was developed after an extensive review of the literature. It is divided into five sections: (1) Socio-demographic factors, (e.g., age, gender, nationality, region of residence, marital status, educational level, employment, monthly income, medical insurance status, and smoking), (2) Current health status, (e.g., presence of comorbidities and perceived physical health), (3) Headache frequency and medication overuse, (4) The Migraine Symptom Severity, and (5) migraine specific quality of life. Subsequently, the survey and its instruments were reviewed by the investigators and a neurologist clinician (n = 4) to ensure clarity and appropriateness of content and ensure content validity.

### 2.5. Survey Validation

Following Institutional Review Board (IRB) approval, the survey underwent pilot testing. Cognitive interviews were conducted with a purposive sample of migraine patients (n = 15), to assess clarity, comprehension, and relevance of each item. Based on feedback, several modifications were made: ambiguous questions were reworded, response options were clarified, and items were adjusted for cultural and linguistic appropriateness. The revised survey was then re-evaluated and finalized through discussion among the study investigators.

## 3. Measures

### 3.1. Study Outcome: Quality of Life

Quality of life in Migraine patients was measured using the Migraine-Specific quality of life Questionnaire, version 2.1 (MSQ 2.1) [20]. It assesses how migraines have impacted a patient’s life over the past four weeks. It covers three domains: role function-restrictive (RR), with seven items measuring how migraines affect a person’s usual social and work life; role function-preventive (RP), with four items measuring how migraines interfere with social and professional activities; and emotional function (EF), with three items measuring headache-related feelings. Participants rate the items on a 6-point Likert scale ranging from “none of the time” to “all of the time. The raw scores for each dimension were calculated by summing the item responses and then rescaling them to a 0–100 range. Higher scores indicate better quality of life.

### 3.2. Independent Variables

The main independent variable was the medication-overuse headache, it was evaluated using the diagnostic criteria outlined in the International Classification of Headache Disorders, 3rd Edition (ICHD-3) [1], based on patient-reported headache frequency and medication use patterns. Information on medication use was obtained through self-report and was not independently verified through prescription records. Assessment of medication overuse headache (MOH) included the number of headache days experienced in the past month, the types of acute medications used to treat headaches, the frequency and duration of medication use, specifically whether it has persisted for more than three months. Acute medications were categorized into simple analgesics (e.g., aspirin, ibuprofen, acetaminophen), combination analgesics (e.g., Acetaminophen-Aspirin-Caffeine), triptans (e.g., sumatriptan, rizatriptan), opioids (e.g., codeine, tramadol), and ergotamines (e.g., ergotamine, dihydroergotamine). Participants were classified as having MOH if they reported experiencing headaches on 15 or more days per month and regular overuse of acute medications (≥15 days per month for simple analgesics or ≥10 days per month for other medications) for at least three consecutive months [1].

Other independent variables included participants’ sociodemographic data (age, gender, nationality, region of residence, marital status, educational level, employment, monthly income, and medical insurance status). Clinical variables included smoking history and the current health status of the participants (the presence and types of chronic health conditions), and perceived physical health (excellent/very good, good, and fair/poor). In addition, migraine severity was evaluated using the Migraine Symptom Severity Scale (MSSS) [21], which is a combined index that incorporates the frequency of seven primary migraine features. These features are: unilateral pain, pulsatile pain, moderate-to-severe pain intensity, routine activities that worsen pain, nausea, photophobia, and phonophobia. Each feature is scored based on its frequency of occurrence, with responses ranging from 1 (never) to 4 (half the time), resulting in a total score between 7 and 28. Higher MSSS scores indicate greater migraine severity, as described in the Chronic Migraine Epidemiology and Outcomes (CaMEO) Study [21].

### 3.3. Confidentiality and Ethical Consideration

Ethical approval was obtained from the institutional review board at King Saud University Medical City (No. E-24-9495). All data collected were entered into a secure electronic database and managed according to the principles of data privacy and confidentiality.

### 3.4. Statistical Analysis

All statistical analyses were conducted using SAS OnDemand for Academics. Descriptive statistics summarized participants’ demographic and clinical characteristics. Categorical variables are presented as frequencies and percentages. Differences in the Migraine-Specific Quality of Life (MSQ) domain scores, Role Restrictive (RR), Role Preventive (RP), and Emotional Function (EF), between groups were assessed using non-parametric tests, because of the non-normal distribution of the MSQ scores; Wilcoxon Rank-Sum test for variables with two categories and the Kruskal–Wallis test for those with three or more categories.

To identify independent factors of MSQ outcomes, multiple linear regression analyses were conducted for each domain (RR, RP, and EF). All linear regression assumptions including linearity, normality of residuals, and homoscedasticity, and multicollinearity using variance inflation factors (VIF) were tested. Given the non-normal distribution of the outcome variable, a generalized linear model (GLM) with a Gamma distribution and log link function was used. This approach is appropriate for positively skewed continuous data and does not require the assumption of normally distributed residuals. Variables included in the models were selected based on clinical relevance and prior literature, as well as their association with the outcome in bivariate analyses. These included MOH status, migraine symptom severity (MSSS grade), headache days per month, health insurance status, use of combination analgesics, and sociodemographic variables. Adjusted regression coefficients (β), 95% confidence intervals (CIs), and *p*-values were reported. Statistical significance was defined as *p* < 0.05.

### 3.5. Sample Size

The required sample size was calculated to address the study’s objectives: estimating the prevalence of MOH in Riyadh, Saudi Arabia, and assessing its relationship with quality of life in migraine patients. For the prevalence analysis, the sample size was determined using the formula n = (Z^2^ × *p* × (1 − *p*))/E^2^, where *p* = 0.25 (assumed prevalence based on prior study [18]), Z = 1.96 (95% confidence level), and E = 0.05 (margin of error). This resulted in a required sample size of 289 participants.

## 4. Results

### 4.1. Participant Demographics

A total of 152 individuals participated in the study, with a predominance of female respondents (76.3%) (Table 1). The age distribution was primarily concentrated in the 40–49 age group (33.6%), followed by those aged 30–39 (30.3%), 18–29 (20.4%), and ≥50 years (15.8%). In terms of education, 61.8% held a university degree, 16.4% had completed postgraduate studies, 13.8% had finished high school, and 7.9% had not completed high school. Regarding employment, 58.6% were employed, 37.5% were not working, and 3.9% were actively seeking employment. Reported monthly income varied, with 35.5% indicating no current income (e.g., students or unemployed). Among the remaining participants, income was distributed nearly equally between those earning 5001–15,000 SAR (28.3%) and more than 15,000 SAR (28.3%), while a small proportion (7.9%) earned less than 5000 SAR. Most participants (64.5%) reported having health insurance, with government insurance being the most common type. The majority of the sample were non-smokers (89.5%), and one-third reported at least one chronic health condition. Self-reported perceived physical health was rated as very good by 31.6% of participants, good by 30.9%, excellent by 17.8%, fair by 13.8%, and poor by 5.9%. The majority of participants had severe migraine (71.7%), while 24.3% had moderate migraine, and 3.9% had mild migraine severity.

### 4.2. Medication Overuse Headache and Medication Usage Patterns

A total of 26 participants (17.1%) met the criteria for Medication Overuse Headache (MOH), while 126 participants (82.9%) did not (Table 2). Regarding medication use, the majority of participants (44.7%) reported using simple analgesics for headache management, while 38.2% used combination analgesics. A smaller proportion of participants (29.6%) used triptans, and 15.1% used opioids. Notably, participants with MOH in this study were significantly more likely to use combination analgesics and opioids compared to those not using these groups of medications. In addition to single-class medication use, overlapping use of acute medications was observed. A notable proportion of patients reported using more than one medication class, including 15.8% using both simple and combination analgesics, 13.2% using simple analgesics and triptans, and 10.5% using combination analgesics and triptans. Furthermore, 7.9% of patients reported using all three classes.

MOH participants were predominantly classified as having severe MSSS (85%), with fewer participants classified with Moderate (15.4%) and none had mild migraine.

### 4.3. Participants’ Mean Migraine-Specific Quality of Life Score by MOH Categories

**Figure 1:**
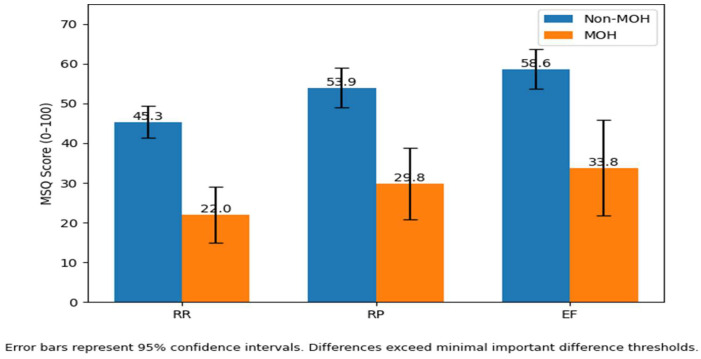
Mean Migraine-Specific Quality of Life (MSQ) Domain Scores (RR, RP, EF) by Medication Overuse Headache (MOH) Status.

According to the MSQ Version 2.1 scoring manual, the Minimal Important Difference (MID), the smallest change considered clinically meaningful, is 3.2 points for RR, 4.6 points for RP, and 7.5 points for EF. When examining the mean MSQ domain scores in our sample (Figure 1), substantial and clinically meaningful differences in migraine-specific quality of life were observed between patients with and without medication overuse headache (MOH). Patients with MOH had markedly lower scores across all MSQ domains (RR: 22.0 vs. 45.3; RP: 29.8 vs. 53.9; EF: 33.8 vs. 58.6). These differences far exceeded the established minimal important difference thresholds (3.2 for RR, 4.6 for RP, and 7.5 for EF), indicating that the observed impairments are not only statistically significant but also clinically meaningful. This corresponds to reductions of more than 20 points across domains, reflecting a substantial deterioration in daily functioning and emotional well-being among patients with MOH.

### 4.4. Factors Associated with Quality of Life from Bivariate Analysis

Bivariate comparisons were conducted to examine associations between partici-pant characteristics and the three domains of the Migraine-Specific Quality of Life: Role-Restrictive (RR), Role-Preventive (RP), and Emotional Functioning (EF) (Table 3). Medication overuse headache (MOH) was strongly associated with significantly lower scores in RR, RP, and EF (all *p* < 0.0001). Use of combination analgesics was associated with lower RR (*p* = 0.004), RP (*p* < 0.001), and EF (*p* = 0.017) scores. Participants with better perceived physical health reported higher quality of life across all three MSQ domains (RR: *p* = 0.015; RP: *p* = 0.012; EF: *p* = 0.007). Migraine symptom severity (MSSS grade) was associated with quality of life across all three MSQ domains (all *p* < 0.001).

Health insurance status was significantly associated with RR (*p* = 0.007) and RP (*p* = 0.002) domains, favoring insured participants. Gender was associated with RP, with males reporting better functioning (*p* = 0.029). Marital status was associated with RP (*p* = 0.039). No statistically significant associations were found between MSQ domain scores and age, smoking status, simple analgesic use, triptan use, opioid use, chronic diseases, region of residence, educational level, monthly income, or employment status (all *p* > 0.05).

### 4.5. Factors Associated with Quality of Life from Adjusted Multiple Linear Regression

Multiple linear regression analysis was performed to identify factors related to migraine-specific quality of life across the RR, RP, and EF domains (Table 4). After adjustment for all covariates, medication overuse headache (MOH) remained independently associated with substantially lower quality of life across all domains. Specifically, MOH was associated with reductions of approximately 11–16 points in MSQ scores, indicating a clinically meaningful decline in functioning and emotional well-being. Migraine symptom severity demonstrated the strongest association with MSQ, with patients experiencing mild symptoms reporting markedly higher scores (increases of up to 30–40 points) compared to those with severe migraine, highlighting the profound impact of symptom burden on daily functioning. Headache days per month was also associated with poorer RR and RP scores. In addition, health insurance was positively associated with RP and RR scores, while use of combination analgesics was linked to lower RP scores. Overall, the models explained a moderate proportion of variance in quality of life (R^2^ ranging from 26.8% to 39.9%), indicating that while MOH and migraine severity are key determinants, other unmeasured factors may also contribute.

## 5. Discussion

This study provides clinic-based evidence on the prevalence of medication-overuse headache (MOH) and its association with migraine-specific quality of life among adults with migraine in Saudi Arabia. The findings indicate that approximately one in six patients met criteria for MOH, a proportion notably higher than estimates reported in both global population-based studies (1–7%) [13,14] and prior Saudi studies (4%) [18,19]. This discrepancy is likely attributable to differences in study design and population characteristics. Unlike community-based surveys, our sample was drawn from a neurology clinic and included patients with clinically confirmed migraine who are more likely to have higher disease severity, and increased exposure to acute medications, all of which elevate the risk of medication overuse.

In terms of patterns of acute medication use, simple analgesics were the most frequently utilized agents in our sample followed by combination analgesics, and triptans; however, opioids were used by a smaller yet clinically meaningful sample. This pattern is consistent with other Saudi studies that have reported frequent reliance on paracetamol and non-steroidal anti-inflammatory drugs, with less widespread use of migraine-specific or prescription-only medications [18,19]. Notably, participants with MOH in this study were significantly more likely to use combination analgesics and triptans. The observed association between frequent use of combination analgesics and poorer outcomes underscores the need for careful prescribing practices. Clinicians may consider limiting overuse, educating patients about risks of medication overuse headache, and prioritizing preventive strategies to reduce reliance on acute combination therapies. Our findings also support guideline-recommended use of triptans as first-line acute therapy while cautioning against routine opioid use, in line with international migraine management guidelines, to minimize risk of dependence and treatment-refractory headache patterns. Taken together, these results highlight actionable considerations for clinicians, including medication selection, patient education, and early implementation of preventive strategies, to optimize outcomes in patients with frequent or refractory migraine.

The key finding of this study is the strong and consistent association between MOH, migraine severity, and reduced migraine-specific quality of life. Patients with MOH demonstrated significantly lower scores across all domains of the Migraine-Specific Quality of Life (MSQ), including role-restrictive, role-preventive, and emotional functioning. Notably, the observed differences exceeded established minimal important difference thresholds, suggesting that these impairments are not only statistically significant but also clinically meaningful [15,16,17].

Although patients with MOH in this study were more likely to have severe migraine symptoms, MOH remained an independent predictor of poorer quality of life even after adjusting for migraine severity, headache frequency, and other covariates. This suggests that MOH may not simply reflect more severe underlying disease but could be associated with poorer patient outcomes. Several mechanisms might underlie this relationship, including central sensitization, reduced responsiveness to treatment, and the psychological burden of frequent headaches and medication overuse; however, these remain speculative in the context of our study. Thus, while MOH appears linked to lower quality of life, the cross-sectional design limits causal inference, and it should be interpreted as a potentially modifiable factor rather than a confirmed independent determinant of worse outcomes.

In addition to MOH, migraine symptom severity emerged as a strong determinant of quality of life across all domains, which is consistent with prior research linking higher symptom burden to greater functional impairment. Headache frequency was also associated with poorer role-related functioning, further emphasizing the cumulative burden of frequent migraine attacks. Interestingly, having health insurance was associated with better quality of life in some domains, possibly reflecting improved access to healthcare services, specialist care, and preventive treatments. This finding underscores the importance of healthcare access in mitigating the burden of chronic migraine. Although several predictors were identified, the explained variance of the models was modest, indicating that a substantial proportion of variability in quality of life remains unaccounted for. This underscores the complex and multifactorial nature of migraine, with contributions from a range of biological, environmental, and psychosocial factors, including potential climatic influences [22]. It also highlights the importance of future research incorporating a broader spectrum of biopsychosocial determinants, including psychological, behavioral, social, and environmental factors, to better understand variability in quality of life outcomes.

### 5.1. Strengths and Limitations

This study has several important strengths. It contributes to the limited evidence examining medication overuse headache and quality of life among migraine patients in Saudi Arabia and uses validated tools, including the MSQ v2.1, the Migraine Symptom Severity Scale, and ICHD-3 criteria, along with clinically confirmed diagnoses of migraine. The inclusion of diverse sociodemographic and clinical variables enabled adjustment for multiple confounders that affect the MSQ outcomes in the linear regression models. The recruitment of a real-world clinical sample from a neurology outpatient setting enhances the clinical relevance and applicability of the findings.

However, this study has several limitations that should be considered. First, its cross-sectional design precludes any inference of causality or temporal relationships. Although MOH was associated with poorer quality of life, the direction of this relationship remains uncertain. It is possible that patients with more severe migraine and greater impairment in quality of life are more likely to overuse acute medications, rather than MOH being the primary cause of reduced quality of life. Therefore, reverse causality cannot be ruled out. Longitudinal studies are needed to better clarify the temporal and causal relationships between MOH and quality of life. In terms of external validity, it is important to note that our sample was drawn predominantly from a specific clinical setting and geographic region. Consequently, the generalizability of these findings to broader populations may be limited. Also, this study relied on self-reported data for key variables, including medication use, which may be subject to recall bias and reporting inaccuracies. Additionally, although MOH was defined according to ICHD-3 criteria, its classification was based on patient-reported information without independent clinical or pharmacy verification. This may have introduced some degree of misclassification. Additionally, psychological factors such as anxiety, depression, and stress known to influence both migraine severity and medication overuse were not assessed in this study; their absence as potential confounders may have affected the observed associations with quality of life. Further, the final sample size (n = 152) was smaller than the initially calculated required sample (n = 289), primarily due to practical challenges in patient recruitment within the study timeframe and the constraints of a busy clinical setting. This reduced sample size may have limited the statistical power of the study and increases the risk of type II error. In addition, given the number of variables included in the regression models, there is a potential risk of overfitting, which may affect the stability and generalizability of the estimates. Therefore, the results should still be interpreted with caution and future studies with larger sample sizes are needed to validate these findings. In addition, the clinic-based recruitment from neurology settings may introduce selection bias, as patients attending specialized clinics are more likely to have more severe or treatment-seeking conditions; this may have led to an overestimation of MOH prevalence and limits the generalizability of the findings to the broader population. Despite these limitations, the study provides valuable insights into the association between medication overuse headache and quality of life among migraine patients and highlights key areas for future research.

### 5.2. Implications

The findings of this study have important clinical and public health implications for migraine management in Saudi Arabia. Medication overuse headache (MOH) is prevalent among adults with migraine, affecting nearly one in six patients in this clinical sample. Regular monitoring of acute medication use is essential to identify patients at risk and prevent the development of MOH. Patients should be educated about the risks of frequent use of analgesics, combination medications, and triptans. Increasing awareness can help patients make informed decisions regarding their treatment and reduce the likelihood of medication overuse. The strong association between MOH and reduced quality of life highlights the need for personalized treatment strategies, including consideration of preventive therapies, to limit reliance on acute medications. In addition, the use of validated instruments such as the MSQ and the Migraine Symptom Severity Scale can help clinicians systematically evaluate functional limitations and emotional impact, enabling comprehensive management plans. The study underscores the need for healthcare policies that support headache clinics, patient education programs, and preventive interventions to reduce the burden of MOH and migraine.

## 6. Conclusions

Medication overuse headache is prevalent and associated with poorer migraine-specific quality of life across role-restrictive, role-preventive, and emotional domains, even after adjusting for headache frequency and severity. Preventive strategies, including early identification of medication overuse, patient education, and appropriate adjustment of treatment regimens, are critical to improving quality of life. Clinicians can consider MOH a modifiable factor in migraine management and integrate screening, counseling, and targeted interventions into routine care. These findings emphasize the importance of the proactive management of medication overuse in migraine patients to preserve daily functioning, emotional well-being, and overall health-related quality of life.

## Figures and Tables

**Table 1:** Demographic Characteristics of Participants (n = 152).

Variable	N (%)
**Age**	
18–29	31 (20.4%)
30–39	46 (30.3%)
40–49	51 (33.6%)
≥50	24 (15.8%)
**Gender**	
Female	116 (76.3%)
Male	36 (23.7%)
**Marital Status**	
Unmarried	59 (38.8%)
Divorced	12 (7.9%)
Married/Widow	81 (53.3%)
**Education**	
Below high school	12 (7.9%)
High school	21 (13.8%)
University	94 (61.8%)
Postgraduate	25 (16.4%)
**Work Status**	
Not working	57 (37.5%)
Seeking job	6 (3.9%)
Working	89 (58.6%)
**Region of Residence**	
Central	142 (93.4%)
Northern	3 (2.0%)
Southern	7 (4.6%)
**Monthly Income**	
No income	54 (35.5%)
<5000 SAR	12 (7.9%)
5001–15,000 SAR	43 (28.3%)
>15,000 SAR	43 (28.3%)
**Health Insurance**	
Yes	98 (64.5%)
No	54 (35.5%)
**Smoking**	
Yes	16 (10.5%)
No	136 (89.5%)
**Chronic Diseases**	
Yes	50 (32.9%)
No	102 (67.1%)
**Perceived Physical Health**	
Excellent	27 (17.8%)
Very good	48 (31.6%)
Good	47 (30.9%)
Fair	21 (13.8%)
Poor	9 (5.9%)
**Headache Days/Month**	
0–4 days	31 (20.4%)
5–9 days	51 (33.6%)
10–14 days	21 (13.8%)
15 days or more	49 (32.2%)
**MSSS Grade**	
Mild	6 (3.9%)
Moderate	37 (24.3%)
Severe	109 (71.7%)

Note: Column percentage are presented in this table. MSSS: Migraine Symptom Severity Scale.

**Table 2:** Medication Overuse Headache and Medication Usage Patterns (n = 152).

Variable		Medication Overuse Headache (MOH)			
	Total Sample	With MOH	No MOH		
	N (%)	N (%)	N (%)	Chi Square	*p* Value
	152 (100.0%)	26 (17.1%)	126 (82.9%)		
**Simple Analgesics**				1.05	0.304
Yes	84 (55.3%)	14 (20.6%)	54 (79.4%)		
No	68 (44.7%)	12 (14.3%)	72 (85.7%)		
**Combination Analgesics**				7.27	0.007
Yes	94 (61.8%)	16 (27.6%)	42 (72.4%)		
No	58 (38.2%)	10 (10.6%)	84 (89.4%)		
**Triptans**					
Yes	45 (29.6%)	12 (26.7%)	33 (73.3%)	4.12	0.042
No	107 (70.4%)	14 (13.1%)	93 (86.9%)		
**Opioids**					
Yes	23 (15.1%)	4 (17.4%)	19 (82.6%)	0.002	0.968
No	129 (84.9%)	22 (17.1%)	107 (82.6%)		
**MSSS Grade**					
Mild	6 (3.9%)	0 (0.0%)	6 (4.8%)	2.64	0.104
Moderate	37 (24.3%)	4 (15.4%)	33 (26.2%)		
Severe	109 (71.7%)	22 (84.6%)	87 (69.0%)		

Note: Raw percentages are presented for medications and column percentages are presented for MSSS: MSSS: Migraine Symptom Severity Scale.

**Table 3:** Bivariate Analysis of Quality of Life Domains (RR, RP, EF) by Variables (n = 152).

Binary Predictors (Wilcoxon)			
Variable	RR *p*-value	RP *p*-value	EF *p*-value
MOH	<0.0001	0.0001	0.0005
Gender	0.372	0.030	0.097
Smoking	0.71	0.454	0.724
Simple analgesics	0.727	0.967	0.629
Combination analgesics	0.004	0.0006	0.017
Triptans	0.363	0.213	0.279
Opioids	0.117	0.123	0.223
Health insurance	0.008	0.002	0.287
Chronic diseases	0.422	0.519	0.4031
**Predictors with ≥3 Categories (Kruskal–Wallis)**			
Predictor	RR *p*-value	RP *p*-value	EF *p*-value
Marital status	0.062	0.039	0.114
Education	0.2	0.397	0.309
Profession	0.933	0.837	0.769
Region of residence	0.837	0.766	0.638
Monthly income	0.16	0.213	0.163
General health	0.015	0.0118	0.007
MSSS Grade	<0.001	<0.001	<0.001
Headache days/month	<0.001	<0.001	0.002

RR: role function-restrictive; RP: role function-preventive, EF: emotional function; MSSS: Migraine Symptom Severity Scale. Note: Differences in the Migraine-Specific Quality of Life (MSQ) domain scores (RR, RP, and EF), between groups were assessed using non-parametric tests, because of the non-normal distribution of the MSQ scores; Wilcoxon Rank-Sum test for variables with two categories and the Kruskal–Wallis test for those with three or more categories.

**Table 4:** Multiple adjusted Linear Regression Results for Quality of Life Domains (RR, RP, EF).

	RR		RP		EF	
Variable	β (95% CI)	*p*-Value	β (95% CI)	*p*-Value	β (95% CI)	*p*-Value
**MOH (Ref = No MOH)**	−11.6 (−21.8, −1.5)	0.024	−12.8 (−24.2, −1.4)	0.026	−16.2 (−29.8, −2.4)	0.020
**Headache Days per Month**	−4.7 (−8.1, −1.4)	0.005	−3.8 (−7.6, −0.1)	0.044	−1.8 (−6.3, 2.7)	0.426
**MSSS Mild (Ref = Severe)**	33.7 (16.6, 50.8)	0.0002	28.5 (9.3, 47.7)	0.003	39.5 (16.3, 62.8)	0.001
**MSSS Moderate (Ref = Severe)**	15.8 (8.1, 23.5)	<0.0001	19.4 (10.7, 28.0)	<0.0001	19.8 (9.3, 30.2)	0.0003
**Health Insurance (Ref = No)**	8.4 (1.4, 15.4)	0.017	13.1 (5.3, 21.0)	0.001	2.7 (−6.7, 12.2)	0.568
**Gender Female (Ref = Male)**	−3.8 (−11.6, 3.9)	0.325	−12.1 (−20.8, −3.4)	0.006	−9.6 (−20.1, 0.9)	0.073
**Combined Analgesics (Ref = No)**	−6.8 (−13.6, 0.1)	0.052	−11.7 (−19.4, −3.9)	0.003	−8.2 (−17.6, 1.0)	0.082

β-coefficient, CI: Confidence Interval; Ref: Reference Group; RR: role function-restrictive; RP: role function-preventive, EF: emotional function.

## Data Availability

The datasets used and/or analyzed during the current study are available from the corresponding author on reasonable request.
